# White Matter Damage in 4,725 Term-Born Infants Is Determined by Head Circumference at Birth: The Missing Link

**DOI:** 10.1155/2018/2120835

**Published:** 2018-02-28

**Authors:** Arne Jensen, Bert Holmer

**Affiliations:** ^1^Campus Clinic Gynecology, Ruhr-University Bochum, Universitätsstr. 140, 44799 Bochum, Germany; ^2^Department of Obstetrics & Gynecology, Klinikum Wilhelmshaven, Friedrich-Paffrath-Straße 100, 26389 Wilhelmshaven, Germany

## Abstract

**Background:**

White matter damage (WMD) is a prime risk factor for cerebral palsy, in part occurring unexplained. Though primarily a problem of preterm infants, there is growing evidence that in large newborns cephalopelvic disproportion and prolonged labor are involved.

**Objective:**

To explore both incidence of and morphometric risk factors for WMD in term-born infants.

**Study Design:**

We related growth variables and risk factors of term-born infants to WMD (61/4,725) using odds ratios of *z*-score bands.

**Results:**

The key result is the novel observation that head circumference is a prime and unique index for WMD in term-born neonates over the whole range of centiles (U-shaped; WMD (%) = 3.1168–0.12797^∗^HC (centile) + 0.0014741^∗^HC^2^; *p* < 0.0001). This suggests different mechanisms for WMD in the lowest and highest *z*-score band. In the latter, cephalic pressure gradients and prolonged labor with preserved neonatal vitality prevail, whereas in the previous one, acute and chronic oxygen deprivation with reduced vitality predominate.

**Conclusions:**

The fact that seemingly healthy term-born neonates are not screened by head imaging, in spite of both large head circumference and prolonged labor, is considered to be the missing link between the insult that escapes diagnosis and the development of unexplained developmental delay and cerebral palsy in childhood.

## 1. Introduction

White matter damage (WMD) is a prime risk factor for cerebral palsy, the most common disability in childhood for which there is no cure at present beyond supportive and occupational care [1, 2]. It is believed that white matter damage is primarily a problem of preterm infants if, for example, periventricular leukomalacia occurs [3]. However, a large cranial ultrasound screening study revealed that a substantial fraction of cases presenting white matter damage (34%–39%) is to be expected in infants born near term [4]. While the etiology of white matter damage in preterm infants is largely related to circulatory changes, for example, systemic hypotension during and/or after asphyxia, and infection, the etiology in term-born infants is less well understood [1, 2]. Though similar mechanisms may apply for preterm and term infants, there is growing evidence that, in large newborns, mechanical problems during delivery resulting in excessive molding and depression of the skull may be involved and perhaps account for unexplained cases of developmental delay or cerebral palsy, despite preserved vitality at birth, as recently observed [5–7]. This led us to evaluate the data from our prospective cranial ultrasound screening study to relate growth variables of term-born infants at birth to white matter damage observed [4]. In this neonatal ultrasound screening study that is the only available trial in which cranial abnormalities were assessed across the whole range of gestational ages (24–43 weeks gestation), the overall incidence of WMD was 0.02%, 0.02%, and 2.6% for periventricular leukomalacia, porencephaly (cystic periventricular leukomalacia), and enlarged lateral ventricles, respectively [4]; however, only cerebral hemorrhage but not WMD was evaluated. We considered a reevaluation of this prospective database mandatory to improve current clinical management of large term-born infants by proposing generous use of cranial imaging after birth because, recently, curative treatments of WMD and cerebral palsy by autologous cord blood stem cells have become available that are most effective when given early after the insult [6, 8]. Furthermore, we wished to discuss the possibility if WMD escaping diagnosis in large term-born infants may perhaps explain in part hitherto unexplained cases of developmental delay and cerebral palsy in childhood. This is of growing importance because neither the general problem of large newborns, prolonged labor, and cephalopelvic disproportion nor the principles of clinical management have changed significantly over the years, but the prevalence has increased dramatically and, unlike previously, a therapeutic option for neonatal WMD has become available [6].

## 2. Materials and Methods

From January 1, 1984, to December 31, 1988, a prospective cranial ultrasound screening study was carried out on all live-born infants on the day of discharge of the mother (5–8 days postpartum) from the Department of Obstetrics and Gynecology at the University of Giessen [4]. Since the time of collection of the data, the technical procedure of cranial ultrasound in newborns and the interpretation of abnormalities have not changed significantly. Infants who had to be transferred to the children's hospital directly postpartum were screened on the neonatal ward. The screening examinations were performed in a single- (investigator-) blinded manner with no access to clinical data by two experienced pediatric radiologists (consultants) only. In case of abnormalities, cranial ultrasound was repeated on a daily basis if required to establish the diagnosis. The protocol was approved by the relevant institutional review board, and neonatal cranial ultrasound was introduced as standard of care. After consent of the mothers, a total of 4,725 term-born neonates (37–43 weeks gestation) were prospectively included in the cranial ultrasound screening, that is, >91% of all term-born infants in that period. All relevant obstetrical data including growth variables and risk factors derived from the birth records were encoded and entered into a database for evaluation.

### 2.1. Cranial Ultrasound

**Figure 1:**
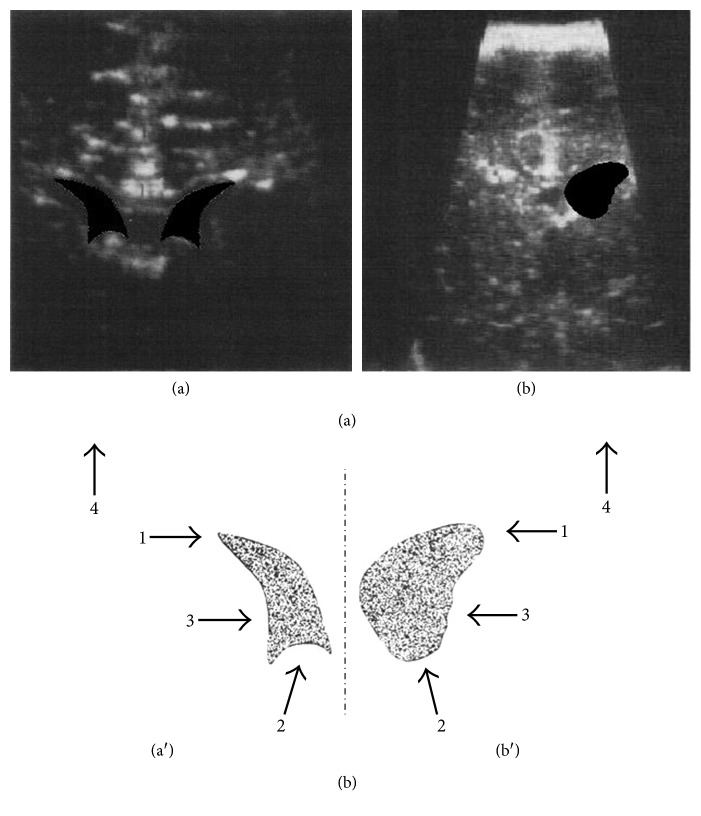
Illustrating cranial sonographic overlay sketch (enlarged) of (a) bilateral physiologic (symmetrical) and (b) unilateral pathologic (asymmetrical) mild enlargements of the lateral ventricles in (a) an infant at 7 weeks and (b) a newborn at 7 days of age. The sketch depicts clear criteria for (a′) physiologic variants (1 = sharp lateral angle, 2 = concave bottom, 3 = smooth inner lining, and 4 = regular echogenicity of the parenchyma in this case) and (b′) pathologic variants (1 = blunt/obtuse lateral angle, 2 = convexly shaped bottom, 3 = irregular inner lining, and 4 = asymmetry, atrophy, and porencephalic cysts as additional findings in this case) of mild enlargements of the lateral ventricle (modified illustration, [9]).

Sonographic examination of the brain was performed on postnatal day 1–30 (median 8 days) through the anterior fontanel in coronal and sagittal sections as previously described [4, 9]. Briefly, high-resolution sector transducers (Siemens Sonoline SL (1984), 5 MHz, Siemens Model RA1 (1980), 7.5 MHz) were used. The coronal section plane ran along the level of the interventricular foramina (Monro) through the lateral ventricle and the third ventricle so that asymmetrical findings such as one-sided lateral ventricle enlargement with or without displacement of the interhemispheric fissure could be diagnosed easily. Sonographically, white matter damage presents as focal or diffuse echodensities, echolucent cysts (porencephaly) in the brain parenchyma, and/or enlarged lateral ventricles [2]. Specific criteria for physiologic and pathologic enlargement of the lateral ventricles, as assessed by diameter, cross-sectional area, and shape, have been developed for this trial in a pilot on 205 clinically unremarkable newborns as described earlier [9]. Pathologic ventricular enlargements were diagnosed threefold, firstly, at and beyond the threshold width of 10 mm in diameter of the lateral ventricle and, secondly, beyond a threshold cross-sectional area of 0.28 cm^2^. Thirdly, to distinguish between physiologic variants (1 = sharp lateral angle, 2 = concave bottom, and 3 = smooth inner lining, i.e., coding “0” in Table 1) and pathologic variants (1 = blunt/obtuse lateral angle, 2 = convexly shaped bottom, and 3 = irregular inner lining, i.e., coding “1” in Table 1) of mild ventricular enlargements, for example, caused by asphyxic and/or hemorrhagic events, the shape of the ventricles was evaluated following the criteria (1–3) depicted in Figure 1. No attempt was made to assess grey matter. The various forms of white matter damage, including “lateral ventricle asymmetry,” “lateral ventricle enlargement,” “lateral ventricle bilateral enlargement” (defined as an enlargement of the lateral ventricle without ventricular asymmetry), “posthemorrhagic hydrocephalus,” “diffuse periventricular leukomalacia,” and “cystic periventricular leukomalacia (porencephaly)” as observed in individual patients (*n* = 61) are listed in Table 1.

### 2.2. Growth Variables and Morphometric Index (MMI)

Growth variables of term-born infants at birth were related to WMD in that *z*-scores of weight (*W*), length (*L*), head circumference (HC), and weight-length ratio (*W*/*L*) were calculated, and significant relations were demonstrated between *z*-score bands as compared with the reference, being particularly close for HC (Table 2). In an effort to improve the risk assessment as part of the clinical management and risk-adapted use of brain imaging techniques, we propose a morphometric index (MMI) that reflects best the white matter damage risk patterns of growth variables (centile), including *W*, *L*, HC, *W*/*L*, and the HC parabola. We averaged the *z*-scores to calculate the MMI as follows: MMI = (*z*-score*W* (centile) + *z*-score*L* (centile) + *z*-scoreHC (centile) + *z*-scoreWeight/Length-ratio (centile) + *z*-score (3.1168–0.12797^∗^HC (centile) + 0.0014741^∗^HC^2^ (centile)))/5. The reference for *z*-score evaluation for lower (centiles <10% and 10 to <25%) and higher (centiles 75 to <90% and ≥90%) bands in each of the growth variables and the MMI was the reference *z*-score value established for the 25 to <75% centile.

### 2.3. Statistical Analysis

Results are presented as means +/− standard deviation (SD). Quantitative and qualitative variables were compared using the chi-square test for contingency tables and ANOVA (using Games–Howell posthoc test to account for multiple comparisons and Welch test for unequal variances) to assess differences in proportions between groups. Odds ratios (OR) were calculated as quantitative measures of the association of white matter damage with given anthropomorphic *z*-score bands, using *z*-scores between −0.67 SD and <0.67 SD (25% to <75%) centile as reference (statistical program: SPSS-24, IBM Corporation, NY, USA). This level was chosen based upon exploratory analysis of all three anthropomorphic measurements (weight (*W*), length (*L*), and head circumference (HC)) showing that this *z*-score band on average had the lowest risk for white matter damage. The analyses were performed in the total term-born population (*n* = 4,725). The relation between head circumference (centile) and the risk for white matter damage (%) was expressed by a 2nd degree polynomial (parabola) curve fit.

## 3. Results

Of 5,799 live-born babies delivered between 1984 and 1988, 5,286 (91.1%) neonates (51.1% male and 48.9% female) underwent cranial ultrasound screening. Of these, 4,725 term-born neonates (including twins) were evaluated (50.8% male and 49.2% female), in 61 (100%) of which white matter damage (WMD) was detected (lateral ventricle enlargement (*n* = 57/61; 93%), lateral ventricle asymmetry (*n* = 29/61; 48%), bilateral enlargement of the lateral ventricle (*n* = 29/61; 48%), posthemorrhagic hydrocephalus (*n* = 3/61; 5%), diffuse periventricular leukomalacia (*n* = 2/61; 3%), cystic periventricular leukomalacia (porencephaly) (*n* = 0/61; 0%)) (Table 1) with no gender-related differences in the overall rate of white matter damage (male 1.4% versus female 1.2%, n.s.).

**Figure 2:**
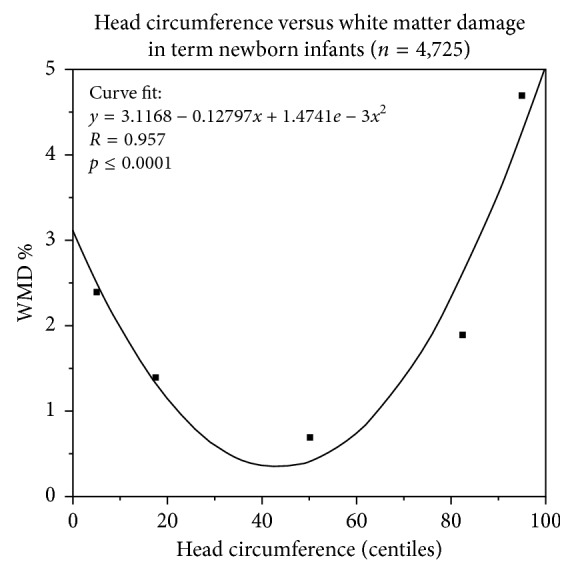
Head circumference determines white matter damage (WMD) assessed by cranial ultrasound in 4,725 term-born neonates. A parabolic correlation curve was fitted to describe the relation between head circumference (HC) in centiles and the risk to develop white matter damage (%): WMD (%) = 3.1168–0.12797^∗^HC (centile) + 0.0014741^∗^HC^2^ (centile) (*r* = 0.957, *p* < 0.0001). Interestingly, from all growth variables at birth, only head circumference showed a close relation to the risk of white matter damage over the whole range of centiles (Table 2).

The odds ratios (OR) for the quantification of the association between growth variables, weight-length ratio, ponderal index, and morphometric index (MMI) of the neonates and white matter damage, according to the centiles of the *z*-score bands, are given in Table 2. Among all term-born neonates, those with the lowest birth weight, length, and head circumference *z*-scores were at increased risk of white matter damage compared with the reference group. Interestingly, only head circumference showed a close relation to the risk of white matter damage over all *z*-score bands in that the odds ratios were significantly different in the <10%, 10 to <25%, 75 to <90%, and ≥90% *z*-score bands as compared with the reference (25 to <75%). This tight U-shaped correlation (parabola, *r* = 0.957, *p* < 0.0001) (Figure 2) between risk for white matter damage (%) and the centiles of head circumference (HC), presenting 2.5, 1.3, 0.4, 2.6, and 4.3% WMD at <10%, 10 to <25%, 25 to <75%, 75 to <90%, and ≥90%, respectively, is of major clinical significance. This unique relation follows the equation (HC-WMD risk formula): WMD (%) = 3.1168–0.12797^∗^HC (centile) + 0.0014741^∗^HC^2^ (centile) and suggests different mechanisms for white matter damage in the lowest and highest *z*-score band, respectively (Figure 2), because in the latter, mechanical pressure gradients along with cephalopelvic disproportion may be involved (Tables 3 and 4). Since, unlike <10% centile, male neonates (347/472 (74%)) significantly outnumber females (124/472 (26%)) in the head circumference *z*-score band ≥90%, in which the risk of white matter damage is high (Table 3), this gender change in proportion might unravel in part as to why cerebral palsy is more prevalent in male than in female children, an unexplained phenomenon hitherto.

From all anthropomorphic indices combining various growth variables at birth, our morphometric index (including growth variables, weight/length ratio, and HC-WMD risk formula) proved to reflect best the risk for white matter damage (%) in both lowest and highest *z*-score bands as compared with head circumference (Table 2). Furthermore, the MMI, which is closely correlated to HC (MMI = 0.692^∗^HC (*z*-score), *r* = 0.851, *p* < 0.001, *n* = 4,724), clearly identified the principal risk factor of WMD in large newborns, that is, unlike in lower *z*-score bands, prolonged or obstructed labor indicating potential cephalopelvic disproportion was significantly increased in the higher MMI *z*-score bands (*p* < 0.001) (Table 3). It is of note that, in the present study, the ponderal index (100^∗^*W*/*L*³), unlike the weight-length ratio (*W*/*L*), does not bear any relation to the risk of white matter damage in term-born neonates.

The obstetrical variables and potential risk factors associated with significant changes in *z*-score bands of the morphometric index as compared with the reference (25 to <75%) are displayed in Tables 3–5. Interestingly, gender proportion changed towards male newborns in higher *z*-score bands >75% in which, conceivingly, the rate of prolonged or obstructed labor, stained amniotic fluid, and pCO_2_ in umbilical arterial blood were also increased. However, variables associated with chronic lack of oxygen and acute asphyxia, for example, growth retardation, pathologic cardiotocography, reduced Apgar scores at 1, 5, and 10 minutes, reduced umbilical arterial blood pH, and increased base excess were significantly more frequent in the <10% *z*-score band along with the number of multiples (largely twins) and presentations other than vertex lies (Table 4). The number of patients and means (SD) of weeks gestation along with growth variables within the MMI <10%, 10 to <25%, 75 to <90%, and ≥90% *z*-score bands (centile) as compared with the reference (25 to <75%) are given in Table 5.

Unlike the risk for white matter damage that was significantly increased in the *z*-score bands <10% and >90%, as was gestosis (Table 3), there were no relations between *z*-score bands of the morphometric index and intra/periventricular hemorrhages, neither overall (IVH/PIVH grade I/II–grade IV) nor individually as grade I/II, grade III, or grade IV. The same holds true for premature rupture of membranes (PROM), bleeding during pregnancy, chorioamnionitis, hypertension or hypotension, and the number of miscarriages (data not shown). Interestingly, maternal fever >38°C during delivery (35/4,725), a prime risk factor for brain damage in preterms [1, 4], did not increase the risk for white matter damage in term-born neonates (data not shown), suggesting a distinct susceptibility of the white matter to inflammation in term as compared with preterm neonates.

From all term-born infants presenting WMD, 38% (23/61) had to be transferred to the neonatal intensive care unit due to perinatal asphyxia as evidenced by lower Apgar scores at one (6.6 (2.5) versus 8.7 (1.1), *p* < 0.001), five (8.2 (2.0) versus 9.8 (0.6), *p* < 0.002), and 10 minutes (8.9 (1.8) versus 9.9 (0.4), *p* < 0.02) as compared with infants not transferred (38/61). Interestingly, the transferred symptomatic infants had a lower weight (3,002 (773) grams versus 3,380 (566) grams, *p* < 0.05) and length (49.3 (4.2) cm versus 51.3 (2.5) cm, *p* < 0.05) at birth as well as evidence for growth retardation as the weight/length ratio was significantly reduced (60.0 (11.6) versus 65.6 (8.9), *p* < 0.05). This favours the view that almost two-thirds (62%) of the term-born infants presenting WMD are large and clinically unremarkable and hence escape routine diagnosis because they are seemingly healthy with normal Apgar scores, thus lacking reasons for transferral. There were no differences between groups as far as mode of delivery is concerned.

## 4. Discussion

This is the first cranial ultrasound screening study for WMD in term-born infants and though the data were collected in the years 1984–1988, the fact that seemingly healthy large newborns escape diagnosis of WMD is of utmost actuality given the rising prevalence of maternal obesity and fetal macrosomia [10]. The new finding that, in an unselected cohort, 1.3% term neonates present WMD was only possible by screening all term-born infants at postnatal day 1–30 because most of the affected infants (62%) are asymptomatic. Importantly, early diagnosis of WMD in these asymptomatic large newborns would improve outcome by maximising the benefit of early intervention, either by timely active (neuro)rehabilitation or by future curative treatment options for WMD using autologous cord blood stem cells that presently are being developed [6, 8, 11].

The cranial ultrasound observation period of 1–30 postnatal days, including follow-up examinations until diagnosis was established, is sufficient to identify WMD with reasonable certainty as demonstrated by brain MRI at a median of 14 days after birth (range 1–60) in a cohort of symptomatic term-born infants in which abnormal T2WI appearance in the white matter (detected in 14% of the cases) was correlated to low Apgar scores at birth, suggesting near birth insults, and severe neurodevelopmental disabilities at 12 months of age [12].

The key result of our study is the novel observation that head circumference at birth is a prime and unique index for increased risk of white matter damage in term-born neonates over the whole range of growth centiles in a U-shaped manner (Table 2, Figure 2). This is an important finding because unlike babies born sick below the 10% centile of growth variables, clinically unremarkable neonates beyond the 75% centile presenting full vitality are usually not examined by imaging of the head and hence escape diagnosis of brain damage with potentially severe consequences for the patients and their families. In particular, since curative treatment options are actually being tested and in part proved to be promising, it is important to establish diagnosis of tentative neonatal brain damage as early as possible to take either full advantage of these treatments or at least start physiotherapy and occupational therapy early [11, 13, 14].

Interestingly, Dahlseng and coworkers investigating growth variables in relation to cerebral palsy in a large epidemiologic study also observed a U-shaped relation for head circumference in the lowest (*p* < 0.001) and highest *z*-score bands (*p* < 0.003) and “total cerebral palsy” (*n* = 398), the group encompassing all subtypes, thus lending strong support to our finding regarding the relation between head circumference and white matter damage in term-born neonates [15]. These authors interpret their epidemiologic findings (in the absence of vitality data, e.g., Apgar scores) by proposing a role for hypoxic-ischemic encephalopathy in large newborns (≥37 weeks' gestation) as a potential cause for cerebral palsy in childhood, particularly in the subtypes of quadriplegic and dyskinetic cerebral palsy. However, we present evidence based on complete clinical data that this pathomechanism causing white matter damage is much more likely to occur in the lowest growth curve centiles (<10%) in which growth retardation and asphyxia prevail (Tables 2, 3–5), whereas white matter damage in large newborns with a head circumference >75% centile occurred in the absence of signs of asphyxia. Our follow-up study in a matched pair design showed that white matter damage is the major determinant of poor performance in psychomotor development at preschool childhood (4.3 SD 0.8 years of age, *n* = 137) in that all three testing domains, intelligence quotient (IQ) (*p* < 0.03), Maze test (*p* < 0.02), and neurologic optimality score (*p* < 0.001), are significantly reduced (Jensen and Neuhäuser, unpublished, EP17171487.6) [16].

Therefore, our findings may elucidate the phenomenon of unexplained cases of developmental delay and cerebral palsy in the pediatric population on the basis of mechanical constraints and pressure gradients squeezing the skull and brain during parturition when a cephalopelvic disproportion is present [6, 7]. Thus, white matter damage in a seemingly healthy population of largely male term-born neonates based on large head circumference appears to be the missing link between the insult that escapes diagnosis and the development of unexplained developmental delay and cerebral palsy in childhood [7, 17]. It is to be hoped that this finding will contribute to prevent in part white matter damage in the future by appropriate obstetrical management of babies presenting large head circumference or, if this is not possible, by early imaging diagnosis and timely therapeutic interventions.

The vast majority of white matter damage detected in this trial was related to enlarged lateral ventricles (93%), either unilateral or bilateral, reflecting subependymal loss of white matter in the periventricular region close to the internal capsule where corticospinal tracts pass (Table 1). We have ascertained that physiologic mild enlargements are identified and distinguished from pathologic mild enlargements of the lateral ventricles in three ways during a pilot on 205 unremarkable newborns using specific criteria for pathology (Figure 1). We determined the ventricular width, the cross-sectional area, and the shape of the ventricle in coronal section planes running through the interventricular foramina to avoid detection of “focal” and/or physiologic enlargements that have been observed in up to 21% of appropriate for gestational age control newborns at the age of 15 years using MRI [18]. However, these authors used control newborns who were large for gestational age (many of them >90% centile), weighing 3,707 (SD 495) grams which is on average 327 grams more than in those seemingly healthy newborns with large head circumferences (>75% centile) that contracted white matter damage in the present study. Interestingly, Skranes and coworkers [18] were unable to detect significant MRI differences in mild ventricular dilatation between small for gestational age term-born infants (*n* = 5/10) and the control group (*n* = 8/14). According to our observations, the likelihood is that the 21% mild ventricular dilatations observed in the “control group” do not constitute a common (“normal”) finding. Rather, they might in part be due to white matter damage in the “control group” based on problems arising from both high birth weights and large head circumference > 90% centile of the newborns that were seemingly healthy and chosen as controls [18].

We present evidence that the mechanisms involved in the development of white matter damage in the lowest and highest head circumference centiles may be fundamentally different in that chronic and acute oxygen deprivation, reduced Apgar scores, umbilical arterial pH, and pathologic heart rate pattern play a major part below the 10% centile, whereas prolonged or obstructed labor and mechanical constraints may be the major determinants of white matter damage beyond the 90% centile (Tables 3–5). The fact that these infants present full vitality as assessed by normal Apgar scores, acid-base balance, and heart rate pattern obscures the excessive risk for white matter damage in these newborns. Though cephalic molding has not been evaluated in this study, the likelihood is that molding was present in these babies with large head circumference because the functional capacity of the pelvis inlet may have been inadequate to allow the fetus to negotiate the birth canal due to cephalopelvic disproportion as evidenced by significantly increased odds ratios for prolonged or obstructed labor (*p* < 0.001) beyond 75% centile *z*-score bands of the morphometric index (Table 3). Therefore, after prolonged labor, a general screening for brain damage in newborns presenting excessive molding of the skull has recently been advocated [6]. Furthermore, we propose that prospective risk management should routinely include sonographic monitoring of head circumference rather than biparietal diameter during late pregnancy, though perhaps technically difficult, to prevent prolonged labor and hence increased risk for white matter damage in babies beyond 75% centile. This seems important because prediction of term infants born large for gestational age, that is, >90% centile, at an earlier stage of gestation using ultrasound and biomarkers have proved to be ineffective [19].

To propose a clinically useful morphometric index (MMI) to easily calculate the risk for white matter damage at given centiles of growth variables, we entered the *z*-scores of *W*, *L*, HC, and the 2nd degree polynomial for HC as well as that of the weight-length ratio into the equation. This combines the advantage of a close relationship between growth variables, particularly head circumference, and odds ratios for white matter damage and the effects of growth retardation on the risk for white matter damage. As shown in Table 2, the provided MMI reflects the significantly increased risk for white matter damage in the lowest as well as in the highest centiles. Thus, by using MMI for white matter damage assessment, unremarkable term-born infants presenting a MMI >90% and <10% centile now can be selected for head imaging as a standard of care to provide a cost-effective basis for potentially curative treatments such as autologous cord blood mononuclear cells that recently have been granted Orphan Drug Designation for the treatment of white matter damage in newborns from the European Medicinal Agency (EMA) [8, 20].

Growth variables like ponderal index and weight-length ratio are commonly used in clinical practice to assess neonatal risk. However, these indices spare the head circumference. Unlike the weight-length ratio, the ponderal index in term-born infants is the only index of all calculated growth variables in the present study that lacked any relation to white matter damage. This is in part at variance to a recent publication on the effects of suboptimal intrauterine environment on late-life cognitive function, in which a lower ponderal index at birth was significantly associated with smaller volumes of total brain and white matter determined by MRI at 75 years of age [21]. Furthermore, in a study on growth variables in relation to cerebral palsy, ponderal index bore some relation to the increased risk of cerebral palsy that was confirmed when the children were 4 years of age [15].

Finally, from an evolutionary point of view, it is evident that the upright gait and hence the prominent promontory along with growing brain volume and general growth acceleration will aggravate the problem of cephalopelvic disproportion and will lead to increased caesarean section rates in the highest *z*-score bands of head circumference in the future to prevent white matter damage. This counterevolutionary process is self-serving because serendipitously the natural selection pressure is being omitted by modern perinatal medicine.

## Figures and Tables

**Table 1:** Various forms of white matter damage (WMD) in term-born neonates at 37–43 weeks gestation diagnosed by cranial ultrasound examination at a median of 8 days (range 1–30 days) after birth.

#	Patient code	Lateral ventricle asymmetry	Lateral ventricle enlargement	Lateral ventricle bilateral enlargement ^∗^	Posthemorrhagic hydrocephalus	Diffuse periventricular leukomalacia	Cystic periventricular leukomalacia (porencephaly)	White matter damage present
1	28	1	1	0	0	0	0	1
2	34	1	0	0	1	0	0	1
3	71	1	1	0	0	0	0	1
4	89	0	1	1	0	0	0	1
5	106	1	1	0	0	0	0	1
6	118	0	1	1	0	0	0	1
7	146	1	1	0	0	0	0	1
8	150	1	1	0	0	0	0	1
9	198	0	1	1	0	0	0	1
10	228	1	1	0	0	0	0	1
11	239	1	1	0	0	0	0	1
12	244	1	1	0	0	0	0	1
13	250	1	1	0	0	0	0	1
14	273	0	1	1	0	0	0	1
15	281	0	1	1	0	0	0	1
16	282	0	1	1	0	0	0	1
17	286	1	1	0	1	0	0	1
18	295	0	0	0	1	0	0	1
19	295	1	1	0	0	0	0	1
20	321	0	1	1	0	0	0	1
21	328	1	1	0	0	0	0	1
22	332	1	1	0	0	0	0	1
23	337	1	1	0	0	0	0	1
24	353	1	1	0	0	0	0	1
25	370	0	1	1	0	0	0	1
26	371	0	1	1	0	0	0	1
27	373	0	1	1	0	0	0	1
28	393	0	1	1	0	0	0	1
29	394	0	1	1	0	0	0	1
30	395	1	1	0	0	0	0	1
31	406	1	1	0	0	0	0	1
32	441	0	1	1	0	0	0	1
33	445	0	1	1	0	0	0	1
34	492	0	1	1	0	0	0	1
35	505	0	1	1	0	0	0	1
36	517	0	1	1	0	0	0	1
37	546	0	1	1	0	0	0	1
38	548	0	1	1	0	0	0	1
39	562	0	1	1	0	0	0	1
40	572	1	1	0	0	0	0	1
41	582	0	1	1	0	0	0	1
42	600	0	1	1	0	0	0	1
43	627	1	1	0	0	0	0	1
44	630	1	1	0	0	0	0	1
45	634	0	0	0	0	1	0	1
46	684	0	1	1	0	0	0	1
47	697	0	1	1	0	0	0	1
48	769	1	1	0	0	0	0	1
49	813	1	1	0	0	0	0	1
50	850	1	1	0	0	0	0	1
51	876	1	1	0	0	0	0	1
52	892	0	1	1	0	0	0	1
53	899	0	0	0	0	1	0	1
54	902	0	1	1	0	0	0	1
55	915	1	1	0	0	0	0	1
56	962	0	1	1	0	0	0	1
57	962	1	1	0	0	0	0	1
58	1,093	1	1	0	0	0	0	1
59	1,118	0	1	1	0	0	0	1
60	1,148	1	1	0	0	0	0	1
61	1,445	0	1	1	0	0	0	1
1–61	Sum	29/61	57/61	29/61	3/61	2/61	0/61	61/61
	%	48	93	48	5	3	0	100

1 = yes; 0 = no; ^∗^Bilateral enlargement of the lateral ventricle is defined as an enlargement of the lateral ventricle without ventricular asymmetry; physiologic variants of mild enlargements of lateral ventricles according to the criteria given in Materials and Methods are coded “0”.

**Table 2:** Odds ratios (OR, *z*-score bands) of white matter damage (WMD) assessed by cranial ultrasound according to anthropomorphic measurements in 4,725 term-born neonates at 37–43 weeks' gestation. Please note that only head circumference (HC) showed a close relation to the risk of white matter damage over all *z*-score bands in that the odds ratios were significantly different in the <10%, 10 to <25%, 75 to <90%, and ≥90% *z*-score bands as compared with the reference (25 to <75%). And also note that the morphometric index (MMI) reflects the increased risk of white matter damage <10% and ≥90% centiles and is calculated as follows: MMI = (*z*-scoreWeight (centile) + *z*-scoreLength (centile) + *z*-scoreHC (centile) + *z*-scoreWeight/Length-ratio (centile) + *z*-score (3.1168–0.12797^∗^HC (centile) + 0.0014741^∗^HC^2^ (centile)))/5.

*z*-score bands (equivalent centiles)																					
Measurement	*n* Newborns screened	*n* WMD (%)	<−1.28 (<10%)	*n*	*n* WMD (%)	*p*	−1.28 to <−0.67 (10 to <25%)	*n*	*n* WMD (%)	*p*	−0.67 to <0.67 (25 to <75%)	*n*	*n* WMD (%)	0.67 to <1.28 (75 to <90%)	*n*	*n* WMD (%)	*p*	≥1.28 (≥90%)	*n*	*n* WMD (%)	*p*
			OR (CI)				OR (CI)				OR			OR (CI)				OR (CI)			
Weight (g)	4,725	61 (1.3)	2.96 (1.51–5.80)	479	14 (2.9)	**0.001**	1.25 (0.58–2.71)	717	9 (1.3)	0.572	Reference	2,284	23 (1.0)	1.11 (0.48–2.60)	772	7 (0.9)	0.807	0.59 (0.26–1.33)	473	8 (1.7)	0.199
Length (cm)	4,725	61 (1.3)	2.70 (1.16–6.28)	257	8 (3.1)	**0.016**	1.15 (0.60–2.19)	1,413	19 (1.4)	0.680	Reference	1,532	18 (1.2)	1.28 (0.59–2.77)	1,082	10 (0.9)	0.540	0.86 (0.34–2.19)	441	6 (1.4)	0.754
Head circumference (cm)	4,725	61 (1.3)	3.48 (1.35–8.98)	255	6 (2.4)	**0.006**	2.10 (1.09–4.04)	1,465	21 (1.4)	**0.023**	Reference	2,328	16 (0.7)	0.34 (0.15–0.76)	504	10 (2.0)	**0.006**	0.14 (0.06–0.34)	173	8 (4.6)	**<0.001**
Morphometric index	4,725	61 (1.3)	0.34 (0.14–0.86)	472	14 (3.0)	**0.001**	0.00 (0.00–4.11)	709	5 (0.7)	0.412	Reference	2,362	26 (1.1)	0.04 (0.00–39.74)	710	4 (0.6)	0.265	1.37 (0.29–6.34)	472	12 (2.5)	**0.011**
Weight-length ratio (*W*/*L*)	4,725	61 (1.3)	2.44 (1.18–5.03)	472	11 (2.3)	**0.013**	0.16 (0.78–3.32)	708	11 (1.6)	0.192	Reference	2,371	23 (1.0)	1.03 (0.44–2.41)	702	7 (1.1)	0.949	0.18 (0.02–2.04)	472	9 (1.9)	0.078
Ponderal index (100^∗^*W*/*L*^3^)	4,725	61 (1.3)	0.84 (0.32–2.17)	470	5 (1.1)	0.717	1.10 (0.54–2.27)	714	10 (1.4)	0.788	Reference	2,361	30 (1.3)	1.10 (0.54–2.27)	714	10 (1.4)	0.788	1.17 (0.51–2.69)	471	7 (1.5)	0.707

Reference value: *z*-score band −0.67 to <0.67 (25th to below 75th centile); All bold *p* values highlight OR (CI) that are significantly different from the reference; the level of significance is given by the probability of error (*p*).

**Table 3:** White matter damage (WMD) and obstetrical variables. Data as mean (SD) and 95% confidence intervals in morphometric index *z*-score bands of 4,725 term-born neonates at 37–43 weeks gestation as compared with the reference (25 to <75%).

	Morphometric index (centile)	*N*	Mean	SD	95% confidence interval		*p*
					Lower limit	Upper limit	
White matter damage present	<10%	472	0.03	0.17	0.01	0.01	0.001
	10 to <25%	709	0.01	0.08	0.00	0.00	n.s.
	25 to <75%	2,362	0.01	0.10	0.00	0.01	Reference
	75 to <90%	710	0.01	0.07	0.00	0.00	n.s.
	90 to <100%	472	0.03	0.16	0.01	0.01	0.011
	Total	4,725	0.01	0.11	0.00	0.01	
EPH gestosis	<10%	472	0.14	0.56	0.09	0.19	<0.001
	10 to <25%	709	0.07	0.25	0.05	0.09	n.s.
	25 to <75%	2,362	0.05	0.22	0.04	0.06	Reference
	75 to <90%	710	0.06	0.23	0.04	0.07	n.s.
	90 to <100%	472	0.11	0.31	0.08	0.13	0.001
	Total	4,725	0.07	0.29	0.06	0.08	
Twins/multiples	<10%	472	0.15	0.35	0.11	0.18	<0.001
	10 to <25%	709	0.05	0.21	0.03	0.06	<0.001
	25 to <75%	2,362	0.01	0.10	0.01	0.01	Reference
	75 to <90%	710	0.00	0.00	0.00	0.00	n.s.
	90 to <100%	472	0.00	0.05	0.00	0.01	n.s.
	Total	4,725	0.03	0.16	0.02	0.03	
Growth retardation (IUGR)	<10%	472	0.25	0.43	0.21	0.28	<0.001
	10 to <25%	709	0.02	0.14	0.01	0.03	n.s.
	25 to <75%	2,362	0.00	0.05	0.00	0.00	Reference
	75 to <90%	710	0.00	0.04	0.00	0.00	n.s.
	90 to <100%	472	0.00	0.00	0.00	0.00	n.s.
	Total	4,725	0.03	0.17	0.02	0.03	
Cardiotocography, pathologic (CTG)	<10%	472	0.23	0.42	0.19	0.26	<0.001
	10 to <25%	709	0.12	0.32	0.09	0.14	n.s.
	25 to <75%	2,362	0.09	0.28	0.07	0.10	Reference
	75 to <90%	710	0.09	0.29	0.07	0.11	n.s.
	90 to <100%	472	0.08	0.28	0.06	0.11	n.s.
	Total	4,725	0.11	0.31	0.10	0.11	
Stained amniotic fluid	<10%	472	0.04	0.21	0.03	0.06	n.s.
	10 to <25%	708	0.04	0.18	0.02	0.05	n.s.
	25 to <75%	2,362	0.04	0.20	0.04	0.05	Reference
	75 to <90%	710	0.09	0.28	0.07	0.11	<0.001
	90 to <100%	472	0.07	0.25	0.04	0.09	n.s.
	Total	4,724	0.05	0.22	0.04	0.06	
Prolonged or obstructed labor	<10%	472	0.08	0.27	0.05	0.10	n.s.
	10 to <25%	709	0.09	0.28	0.07	0.11	n.s.
	25 to <75%	2,362	0.11	0.31	0.10	0.12	Reference
	75 to <90%	710	0.17	0.38	0.15	0.20	<0.001
	90 to <100%	472	0.21	0.41	0.17	0.24	<0.001
	Total	4,725	0.12	0.33	0.11	0.13	
Mode of delivery	<10%	472	1.47	0.83	1.39	1.54	0.001
	10 to <25%	709	1.38	0.86	1.31	1.44	n.s.
	25 to <75%	2,362	1.30	0.72	1.27	1.33	Reference
	75 to <90%	710	1.40	0.79	1.34	1.45	0.033
	90 to <100%	472	1.42	0.74	1.36	1.49	0.013
	Total	4,725	1.35	0.77	1.33	1.38	
Presentation	<10%	471	1.23	0.64	1.18	1.29	<0.001
	10 to <25%	708	1.14	0.51	1.10	1.18	n.s.
	25 to <75%	2,360	1.10	0.43	1.08	1.11	Reference
	75 to <90%	710	1.07	0.38	1.05	1.10	n.s.
	90 to <100%	472	1.07	0.38	1.04	1.11	n.s.
	Total	4,721	1.11	0.46	1.10	1.12	
Gender	<10%	472	1.60	0.49	1.55	1.64	0.013
	10 to <25%	708	1.57	0.50	1.53	1.61	n.s.
	25 to <75%	2,358	1.52	0.50	1.50	1.54	Reference
	75 to <90%	709	1.42	0.49	1.39	1.46	<0.001
	90 to <100%	472	1.26	0.45	1.22	1.30	<0.001
	Total	4,719	1.49	0.50	1.48	1.51	

**Table 4:** Neonatal variables, Apgar scores, and acid-base balance as mean (SD) and 95% confidence intervals in morphometric index *z*-score bands of term-born neonates at 37–43 weeks gestation as compared with the reference (25 to <75%).

	Morphometric index (centile)	*N*	Mean	SD	95% confidence interval		*p*
					Lower limit	Upper limit	
Apgar score_1′	<10%	471	8.41	1.38	8.28	8.53	<0.001
	10 to <25%	709	8.76	1.10	8.68	8.84	0.032
	25 to <75%	2,359	8.88	0.84	8.85	8.92	Reference
	75 to <90%	710	8.85	0.84	8.79	8.92	n.s.
	90 to <100%	470	8.79	0.90	8.70	8.87	n.s.
	Total	4,719	8.80	0.96	8.78	8.83	
Apgar score_5′	<10%	470	9.55	0.95	9.46	9.63	<0.001
	10 to <25%	709	9.79	0.65	9.74	9.84	0.033
	25 to <75%	2,358	9.87	0.50	9.85	9.89	Reference
	75 to <90%	710	9.88	0.43	9.85	9.91	n.s.
	90 to <100%	470	9.81	0.63	9.76	9.87	n.s.
	Total	4,717	9.82	0.59	9.80	9.84	
Apgar score_10′	<10%	471	9.83	0.59	9.78	9.89	<0.001
	10 to <25%	709	9.94	0.31	9.92	9.97	n.s.
	25 to <75%	2,360	9.97	0.23	9.96	9.98	Reference
	75 to <90%	710	9.97	0.20	9.96	9.99	n.s.
	90 to <100%	470	9.96	0.26	9.94	9.99	n.s.
	Total	4,720	9.95	0.30	9.94	9.96	
pH, umbilical arterial blood (UAB)	<10%	466	7.27	0.09	7.26	7.28	<0.001
	10 to <25%	690	7.28	0.07	7.28	7.29	n.s.
	25 to <75%	2,318	7.29	0.07	7.28	7.29	Reference
	75 to <90%	701	7.28	0.06	7.28	7.29	n.s.
	90 to <100%	467	7.28	0.07	7.27	7.28	n.s.
	Total	4,642	7.28	0.07	7.28	7.28	
pCO_2_ (UAB) (mmHF)	<10%	464	45.5	10.2	44.6	46.5	n.s.
	10 to <25%	688	44.2	8.2	43.6	44.8	n.s.
	25 to <75%	2,305	44.6	8.3	44.2	44.9	Reference
	75 to <90%	700	44.8	7.7	44.2	45.4	n.s.
	90 to <100%	462	45.9	8.0	45.2	46.6	0.015
	Total	4,619	44.8	8.4	44.5	45.0	
Base excess (UAB) (mmol/*L*)	<10%	464	7.4	4.5	7.0	7.8	0.009
	10 to <25%	683	7.0	3.9	6.7	7.3	n.s.
	25 to <75%	2,297	6.7	3.7	6.6	6.9	Reference
	75 to <90%	696	7.0	3.9	6.7	7.3	n.s.
	90 to <100%	459	6.9	3.9	6.5	7.2	n.s.
	Total	4,599	6.9	3.9	6.8	7.0	

**Table 5:** Weeks gestation and growth variables as mean (SD) and 95% confidence intervals in morphometric index *z*-score bands of 4,725 term-born neonates at 37–43 weeks' gestation as compared with the reference (25 to <75%).

	Morphometric index (centile)	*N*	Mean	SD	95% confidence interval		*p*
					Lower limit	Upper limit	
Weeks gestation	<10%	472	38.67	1.4	38.5	38.8	<0.001
	10 to <25%	709	39.24	1.3	39.1	39.3	<0.001
	25 to <75%	2,362	40.00	1.1	40.0	40.0	Reference
	75 to <90%	710	40.39	1.0	40.3	40.5	<0.001
	90 to <100%	472	40.58	0.9	40.5	40.7	<0.001
	Total	4,725	39.87	1.3	39.8	39.9	
Weight (g)	<10%	472	2,508	274	2,483	2,532	<0.001
	10 to <25%	709	2,951	136	2,941	2,961	<0.001
	25 to <75%	2,362	3,409	236	3,400	3,419	Reference
	75 to <90%	710	3,783	211	3,768	3,799	<0.001
	90 to <100%	472	4,211	280	4,186	4,236	<0.001
	Total	4,725	3,387	500	3,372	3,401	
Length (cm)	<10%	472	47.4	2.0	47.2	47.6	<0.001
	10 to <25%	709	49.6	1.3	49.5	49.7	<0.001
	25 to <75%	2,362	51.6	1.6	51.5	51.6	Reference
	75 to <90%	710	53.2	1.6	53.1	53.3	<0.001
	90 to <100%	472	54.6	1.8	54.4	54.8	<0.001
	Total	4,725	51.4	2.5	51.3	51.5	
Head circumference (cm)	<10%	472	32.9	1.3	32.8	33.1	<0.001
	10 to <25%	709	33.8	0.8	33.8	33.9	<0.001
	25 to <75%	2,362	34.8	0.9	34.7	34.8	Reference
	75 to <90%	710	36.1	0.6	36.1	36.2	<0.001
	90 to <100%	472	37.1	0.9	37.0	37.2	<0.001
	Total	4,725	34.9	1.4	34.8	34.9	
Weight-length ratio (*W*/*L*)	<10%	472	52.89	5.07	52.43	53.34	<0.001
	10 to <25%	709	59.60	3.32	59.35	59.84	<0.001
	25 to <75%	2,362	66.13	4.31	65.95	66.30	Reference
	75 to <90%	710	71.16	4.05	70.87	71.46	<0.001
	90 to <100%	472	77.15	4.64	76.73	77.57	<0.001
	Total	4,725	65.68	7.60	65.46	65.90	
PI (100^∗^*W*/*L*³)	<10%	472	2.36	0.26	2.34	2.39	<0.001
	10 to <25%	709	2.44	0.23	2.42	2.45	<0.001
	25 to <75%	2,362	2.49	0.23	2.49	2.50	Reference
	75 to <90%	710	2.53	0.24	2.51	2.54	<0.001
	90 to <100%	472	2.60	0.24	2.58	2.62	<0.001
	Total	4,725	2.49	0.24	2.48	2.49	
